# The Utilisation of Acrylamide by Selected Microorganisms Used for Fermentation of Food

**DOI:** 10.3390/toxics9110295

**Published:** 2021-11-05

**Authors:** Katarzyna Petka, Łukasz Wajda, Aleksandra Duda-Chodak

**Affiliations:** 1 Faculty of Food Technology, University of Agriculture in Krakow, ul. Balicka 122, 30-149 Krakow, Poland; katarzyna.petka@urk.edu.pl; 2 CDC Poland sp. z o.o., ul. Zagnańska 153, 25-563 Kielce, Poland; lukasz.wajda8@gmail.com

**Keywords:** lactic acid bacteria, probiotic, yeast, acrylamide, amidase, response surface methodology

## Abstract

Acrylamide (AA) present in food is considered a harmful compound for humans, but it exerts an impact on microorganisms too. The aim of this study was to evaluate the impact of acrylamide (at conc. 0–10 µg/mL) on the growth of bacteria (*Leuconostoc mesenteroides, Lactobacillus acidophilus* LA-5) and yeasts (*Saccharomyces cerevisiae*, *Kluyveromyces lactis* var. *lactis*), which are used for food fermentation. Moreover, we decided to verify whether these microorganisms could utilise acrylamide as a nutritional compound. Our results proved that acrylamide can stimulate the growth of *L. acidophilus* and *K. lactis*. We have, to the best of our knowledge, reported for the first time that the probiotic strain of bacteria *L. acidophilus* LA-5 is able to utilise acrylamide as a source of carbon and nitrogen if they lack them in the environment. This is probably due to acrylamide degradation by amidases. The conducted response surface methodology indicated that pH as well as incubation time and temperature significantly influenced the amount of ammonia released from acrylamide by the bacteria. In conclusion, our studies suggest that some strains of bacteria present in milk fermented products can exert additional beneficial impact by diminishing the acrylamide concentration and hence helping to prevent against its harmful impact on the human body and other members of intestinal microbiota.

## 1. Introduction

Changes in the standard of living, eating habits, and an increased awareness of the impact of nutrition on human health have resulted in more and more consumers paying attention not only to the nutritional value of food products; consumers are increasingly convinced that food affects their health, enables the prolongation of life and well-being, and may also prevent non-transmissible chronic diseases [1]. Hence, each year we can find more types of the so-called functional food on the market. It is estimated that 60–80% of functional products available on the market are probiotics [2]. It is generally accepted that such foodstuffs should contain a minimum concentration of 10^6^ cfu (colony forming units) of probiotics per mL or g. However, many studies report that the minimum dose of 10^7^ cfu/g (or mL) probiotic bacteria in food is recommended. Moreover, a total of some 10^8^ to 10^9^ probiotics must be consumed each day for the beneficial impact to the consumer [3,4,5,6,7].

Dairy products contain a variety of lactic acid bacteria (LAB) and yeasts, which are responsible for the organoleptic and health-promoting properties of these products. Among the products containing a lot of such microorganisms, we can distinguish firstly natural yoghurts, kefirs, ripening cheeses, fermented and acidophilic milk and kumis. The microorganisms present in them are bacteria strains belonging mainly to the species of lactobacilli, bifidobacteria and dairy streptococci such as *Streptococcus salivarius* subsp. *thermophilus*. Fermented milk drinks also contain yeasts of various types and species (e.g., *Kluyveromyces marxianus (Candida kefyr), K. lacits, Saccharomyces cerevisiae, S. exiguus, S. omnisporus*) [1].

Acrylamide (AA) is formed in food mainly by the reaction of free asparagine with reducing sugars (especially fructose and glucose) during the Maillard reaction. However, it can also be produced by other pathways (e.g., the acrolein pathway) [8]. It means that the high temperatures (above 120 °C) used during the thermal processing of food, in addition to imparting flavour and aroma, may lead to the formation of harmful substances in the product, such as acrylamide. The highest acrylamide concentrations are reported in potato crisps, potato chips, instant coffee, biscuits, and French fries, exceeding even 1000 µg/kg. However, the maximum acrylamide concentration reported in some samples of food commodities was above 8000 µg AA/kg [9]. The World Health Organisation (WHO) and Food and Agriculture Organisation of the United Nations (FAO) estimate that the mean human dietary exposure to acrylamide at the national level varies between 0.2 and 1 μg AA/kg-bw per day for the general adult population. However, it can exceed 4.0 μg AA/kg-bw per day for consumers with a high dietary exposure [9]. On the basis of the laboratory tests in animals, the International Agency for Research on Cancer [10] assigned acrylamide to the group of compounds “probably carcinogenic to humans”, while according to the U.S. EPA [11], acrylamide at a concentration of 0.8 µg/L causes a cancer risk of 1: 10,000.

Various experiments have been performed to assess the acrylamide impact on live organisms covering both in vitro and in vivo animal models as well as human studies on AA exposure. It has been proven that acrylamide exerts neurotoxic [12,13], immunotoxic [14], genotoxic [15], cytotoxic [16], and carcinogenic [17,18] impact on the animal or human cells and organisms [13,19]. However, as it does not exert a mutagenic effect in bacterial cells, it was concluded that the carcinogenic activity of acrylamide is related rather to glycidamide (GA), a metabolite of acrylamide formed in mammalian cells [8,20].

Some studies have shown that selected strains of *Lactobacillus* spp. can alleviate the toxicity of acrylamide against rats [21,22]. However, when the studies have looked at the detailed mechanism of this action, they have shown that it consists in removing acrylamide from the solution by its physical binding to the bacterial cell wall [23]. The significant role of peptidoglycan structure, especially the structure of teichoic acids and the contents of four amino acids (alanine, aspartic acid, glutamic acid, and lysine) was also studied in details by Serrano-Niño et al. [24] and Zhang et al. [25].

It is known that some microbial enzymes can catalyse the acrylamide degradation, and the most important among them are amidases [8]. Amidases are enzymes (EC. 3.5.1.4) that occur ubiquitously in nature. They are characterised by a broad spectrum of catalysed reactions and can use various chemicals as a substrate. They play an important role in the bacteria proliferation of cells and separating young cells (e.g., amidases AmiA, AmiB and AmiC synthetized by *E. coli*). However, the substrate for those enzymes is peptidoglycan [26]. There are no reports describing whether bacteria producing amidases, which degrade amide bonds in peptidoglycan, are able to degrade acrylamide as well. However, this cannot be excluded. There are also other enzymes that operate similarly. One of them is N-acetylmuramidase (AcmA, EC 3.5.1.28) synthesised by various strains of *Lactococcus lactis* demonstrating autolysin activity, that has been already applied in cheese maturation. The result of cell autolysis is the release of bacterial peptidases leading to the formation of peptides and amino acids that provide the desired aroma and flavour of cheese [27,28].

The participation of intestine microbiota in chemical changes of AA has not been investigated yet. However, it has been shown that various bacteria, including those that naturally occur in the human gut or those that are delivered with food, might degrade that compound. The ability of synthesising amidases by bacteria present in digestive tract, such as *Escherichia coli, Enterococcus faecalis, Bacillus clausii, Helicobacter pylori* [8] indicates the possible existence of an undiscovered mechanism of acrylamide degradation in the human gut.

In our previous work we demonstrated that acrylamide could influence the viability of beneficial intestinal bacteria from *Lactobacillus* genus [29]. First of all, we demonstrated that the tested lactic acid bacteria strains were tolerant to acrylamide even at high concentrations (up to 1 g/mL) and the growth of *Lactobacillus plantarum, L. brevis*, and *Lactococcus lactis* subsp. *lactis*, as well as probiotic strain *Lactobacillus acidophilus* LA-5, was more intense in the presence of acrylamide at high concentration than in medium with limited accessibility of carbon and nitrogen compounds. It was concluded that acrylamide had no toxic impact on LAB, and that some strains probably could utilise acrylamide as a source of carbon and nitrogen if they lacked them in the environment/medium. Moreover, the viability of bacteria was modulated by acrylamide, the viability of probiotic strain *L. acidophilus* LA-5 increased, that of *L. plantarum* decreased, while *L. brevis* was the less sensitive.

Based on the above, the aim of the present study was to check whether the microorganisms that are often used for fermentation of milk products (lactic acid bacteria and yeasts), which growth was stimulated by acrylamide, are able utilise it. Moreover, we checked if the acrylamide was decomposed by amidases by evaluating the concentration of released ammonia.

## 2. Materials and Methods

### 2.1. Microorganisms and Materials

Pure cultures of yeasts that occur in fermented food products (*Saccharomyces cerevisiae* DSM 70478, *Kluyveromyces lactis* var. *lactis* DSM 70799) and lactic acid bacteria (*Streptococcus salivarius* subsp. *thermophilus* DSM 20617, *Leuconostoc mesenteroides* subsp. *cremoris* DSM 20346) were purchased from Leibniz Institute DSMZ-German Collection of Microorganisms and Cell Cultures GmbH (Braunschweig, Germany), while the probiotic strain *Lactobacillus acidophilus* LA-5 was provided by Chr. Hansen (Hørsholm, Denmark). All microbiological media and components of growth media were purchased from BioMaxima (Lublin, Poland) unless otherwise stated. For the experiments the low-carbon and low-nitrogen medium, a Maximum Recovery Diluent (MRD) was used. MRD was composed of 0.45% (*w*/*v*) of bacteriological peptone (as the only source of carbon and nitrogen in this medium) and 0.45% NaCl (*w*/*v*) (POCh, Gliwice, Poland). YM agar was prepared by dissolving yeast extract (3.0 g/L), malt extract (3.0 g/L), peptone from soybeans (5.0 g/L), glucose (10.0 g/L), agar (15.0 g/L) and chloramphenicol (0.1 g added after sterilisation) in distilled water. All media were sterilised using a Microjet Microwave Autoclave (process parameters: 135 °C, 80 s, 3.6 bar; Enbio Technology Sp. z o.o., Gdynia, Poland). Acrylamide (purum, ≥98%, GC) was provided by Sigma-Aldrich (Sp. z o.o, Poznan, Poland).

### 2.2. Bacteria and Yeasts Preparation

All microorganisms were cultured 24 h before experiments in optimal media and temperatures. Yeasts were grown at 28 °C in a Sabouraud broth supplemented with chloramphenicol (100 mg/L) in 100 mL flasks on the rotary shaker (Orbit 1000, Labnet International Inc., Edison, NJ, USA) at 120 rpm. Bacterial strains were grown in MRS broth at 37 °C (*L. acidophilus* LA-5, *S. salivarius* subsp. *thermophilus*) or 30 °C (*L. mesenteroides* subsp. *cremoris*). Just before experiments the cultures of microorganisms were centrifuged and rinsed (as described in Section 2.3), and the resulting pellets were resuspended in MRD so as to obtain the bacterial/fungal suspensions containing 10^7^ cfu/mL. For that purpose, the optical density of microorganisms’ solution was measured by Den-1B densitometer (Biosan, Latvia) and expressed in McFarland units. The bacteria and yeast concentrations were calculated from the relationship established during calibration.

### 2.3. Measurement of Optical Density of Bacterial and Yeasts Suspension: Calibration

To tubes containing 5 mL of sterile MRS medium, a volume of 0.1 mL of 24-h liquid bacterial culture was added, the contents were mixed, and the tubes were incubated for 24 h at the optimal temperature for the tested strain (listed above). After incubation, bacterial cultures were centrifuged at 194× *g* for 15 min at room temperature (MPW-35JR centrifuge, MPW MED Instruments, Warsaw, Poland), and the supernatant was discarded. The pellets were rinsed twice by mixing with 5 mL of sterile distilled water followed by centrifugation (parameters as above) to remove the residues of growth media. The resulting pellets were resuspended in MRD so as to obtain an optical density of the bacterial suspensions equal to McFarland standard 1.0 when measured by a densitometer (in five replicates). Then, serial 10-fold dilutions were made in MRD, and 1 mL of subsequent dilution was inoculated in the MRS medium with pour plate method (each dilution was inoculated in triplicate). After 72 h of incubation at an optimal temperature in aerobic condition, bacterial colonies were counted, and the mean bacterial cell density (expressed in cfu/mL) from replicates was calculated for each tested strain. The relationship between the optical density of McFarland =1 and bacterial cell density was determined. None of strains used in the current study was strict anaerobe, so the pour plate method used for inoculation was sufficient to provide them proper growth conditions. Exactly the same experiments were carried out for tested yeasts strains, except that Sabouraud broth and Sabouraud agar were used instead of MRS, the spread method was used for cultivation instead of pour plate method, and the incubation temperature was 28 °C.

The relationships obtained for individual strains were as follows (the optical density value in McFarland units that corresponds to the cell concentration of 10^7^ cfu/mL): *L. acidophilus* LA-5 (0.22), *L. mesenteroides* (0.03), *S. thermophilus* (0.03), *K. lactis* (1.23) and *S. cerevisiae* (7.89). In the case of *S. thermophilus* and *L. mesenteroides* optical density was firstly adjusted to 0.3 and then diluted 10-fold.

### 2.4. Preparation of Acrylamide “Stock” Solution

A concentrated (100 mg/L) aqueous solution of acrylamide was sterilised by membrane filtration (pore φ = 0.22 µm; PES Millex-GP, Bionovo, Poland) and diluted (if needed) with sterile distilled water to obtain “stock” solutions of acrylamide (concentrations: 25.0, 50.0, and 75.0 mg/L).

### 2.5. The Impact of Acrylamide Concentration on Microbial Growth

The impact of acrylamide on tested microorganisms was determined in liquid cultures. Control samples were prepared by mixing 8 mL of MRD, 1 mL of microbial suspension (containing 10^7^ cfu/mL) and either 1 mL of sterile water (negative control, C_neg_) or 1 mL of MRD (positive control, C_pos_). Based on the above, the calculated amount of microorganism at the beginning of experiment was 10^6^ cells/mL. Test samples contained 8 mL of MRD, 1 mL of microbial suspension (10^7^ cfu/mL) and 1 mL of adequate acrylamide “stock” solution, so that the final concentration of acrylamide was 2.5, 5.0, 7.5, and 10.0 µg/mL. Therefore, it was possible to track how growing amount of additional carbon and nitrogen source influence the microbial growth (starting from the lower amount in negative control, through the samples with growing acrylamide concentration up to positive control). Experimental variants were incubated at temperatures optimal for the growth of each tested strain, i.e., 28 °C (both yeast strains), 30 °C (*L. mesenteroides*), 37 °C (*L. acidophilus* LA-5 and *S. thermophilus*). Separate sets of experimental variants were prepared for each time interval (0, 24 h and 48 h) for each microorganism. The number of microorganisms at the beginning (time 0 h) and after 24 h and 48 h was enumerated by the spread plate method on MRS agar (for LAB) or YM agar (yeast), wherein 1 mL of 10-fold serially diluted samples were inoculated on the plates. Plates were incubated at optimal temperatures for 72 h and colonies were counted. The experiment was performed in five replicates.

An acrylamide concentration range up to 10 µg/mL was chosen as the value that represents the highest acrylamide level possible to achieve in the human intestine. For calculations, the highest concentrations of acrylamide in food reported in the scientific publication (9728 µg/kg [30]) was taken into account as well as the mean volume of all digestive juices (8–10 L/day) produced by the human gastrointestinal tract.

### 2.6. Assessment of the Impact of pH and Temperature on Microbial Growth in the Presence of Acrylamide

Based on the results of the experiment described in Section 2.5, where the presence of acrylamide seemed to stimulate the growth of *L. acidophilus* LA-5 and *K. lactis*, these two microorganisms were selected for further studies. A set of tubes with MRD with an appropriate acrylamide concentration (7.5 µg AA/mL for *L. acidophilus* LA-5 and 10 µg AA/mL for *K. lactis* as the level causing the highest growth stimulation) were prepared and pH in the media was adjusted with 0.1 M HCl or 0.1 M Na_2_CO_3_ ranging from 2.0 to 9.0 (representing the pH conditions in various parts of the human gut). Then 1 mL of microbial suspension (10^7^ cfu/mL) prepared as described in Section 2.3. was added to 9 mL of MRD with corrected pH, incubated and cells count was enumerated as above. Controls for each strain was constituted of 9 mL of MRD (without AA) adjusted to the adequate pH value and inoculated with 1 mL of microbial suspension. They were prepared to exclude pH conditions that inhibited microbial growth. Each experiment was performed in five replicates. Basing on the results, pH range 4–9 was selected for *K. lactis* and pH range 5–8 for *L. acidophilus* LA-5.

After selecting the optimum pH for microbial growth, each strain was incubated at such pH and at three different temperatures: 4 °C (corresponding to the cooling conditions), 20 °C (room temperature) and 37 °C (the temperature of the human body, e.g., in the human intestine). Enumeration of microorganisms was carried out as above. Each experiment was performed in five replicates.

### 2.7. Assessment of the Microorganism’s Ability to Acrylamide Utilisation

On the basis of the above experiments the microorganism which growth had been stimulated by acrylamide was chosen to check whether they are able to decompose acrylamide by amidase production and utilise it for their growth. The amidase activity was detected by the assessment of the ammonia released during such reaction using a commercial kit (Ammonia Assay Kit, cat. AA0100, Sigma Aldrich, St. Louis, MI, USA). The experiments were carried out in MRD with the addition of acrylamide at concentration of 7.5 µg/mL for *L. acidophilus* LA-5 and 10.0 µg/mL for *K. lactis*. The medium was inoculated with 1 mL of microbial suspension (10^7^ cells/mL) prepared from the 24-h culture as described earlier) and incubated for 48 h at 37 °C (bacteria) or 28 °C (yeast). Separate sets of experimental variants were prepared for each time interval (0, 24 h and 48 h) for each microorganism. At the beginning (time 0 h) and after 24 h and 48 h of incubation the medium was collected and the ammonia concentration was determined spectrophotometrically at 340 nm using commercial kit. In this assay ammonia reacts with α-ketoglutaric acid and reduced nicotinamide adenine dinucleotide phosphate (NADPH) in the presence of L-glutamate dehydrogenase to form L-glutamate and oxidised nicotinamide adenine dinucleotide phosphate (NADP+). The decrease in absorbance at 340 nm, due to the oxidation of NADPH to NAPD+, is proportional to the ammonia concentration. L-Glutamate dehydrogenase reacts specifically with ammonia.

Control samples contained 9 mL of MRD without acrylamide inoculated with 1 mL of the same microbial suspension as a test sample. The ammonia concentration in test samples was compared to the control. The cell free controls were also included in the experiments. We tested ammonia concentration in medium without microorganism before and after the experiments (to evaluate whether ammonia is not present in medium or is not generated in other processes) as we also made controls without acrylamide (to check whether ammonia is formed from the MRD components). We also checked the impact of various pH and temperature on ammonia level in samples without acrylamide and microorganisms. Each experiment was performed in 5 replicates.

### 2.8. Response Surface Methodology (RSM)

The response surface methodology (RSM) can be used to evaluate the relative significance of several factors in the presence of complex interactions. In our study, RSM was carried out according to the procedure described by Lenth [31] and it was applied to evaluate the effect of three various parameters on acrylamide degradation rate measured as the concentration of ammonia released by microorganisms. The experimental design was based on the Box-Behnken design matrix. Seven experimental runs were carried out and the experimental design, variables, coded and decoded levels, and responses are listed in details in Section 3. Results and Discussion. We considered three variables that proved to be significant for the growth of *L. acidophilus* LA-5 which were: pH, time, and temperature. The variable that was measured as the outcome was the concentration of ammonia (µg/mL). The obtained model was prepared according to the Equation (1), which expresses the relationship between the predicted response and independent variables in coded values:

(1) $$Y=\beta_{0}+\sum_{i=1}^{3}\beta_{i}X_{i}+\sum_{i=1}^{3}\beta_{ii}X_{i}^{2}+\sum_{i<j}\beta_{ij}X_{i}X_{j}$$

where *Y* is the estimated response, *β*_0_, *β_i_*, *β_ii_* and *β_ij_* are the regression coefficients for intercept, linearity, square and interaction, respectively, while *X_i_* and *X_j_* are the independent coded variables.

### 2.9. Basic Statistics and Principal Component Analysis (PCA)

All experiments described in the current paper were carried out in five replicates. The results are shown as arithmetic mean ± standard deviation (SD). The normality of distribution was assessed by the Shapiro–Wilk test and two-way analysis of variance (ANOVA) with Tukey’s honest significant difference (HSD) posthoc test was used to compare mean values and determine the significance of differences. A *p*-value < 0.05 was considered statistically significant.

Principal component analysis (PCA) enables the selection of process parameters which are mostly correlated with values measured as outcomes of the experiments (in our case–the cells number). It combines the benefits of the analysis of both variance and correlation. When we deal with complex dataset considering many variables, it enables the selection of only those variables that have a significant impact on examined processes. In our study, principal component analysis with varimax rotation was applied to assess correlations among variables. All statistical analyses were carried out using R: A language and environment for statistical computing, version 3.5.0 (Foundation for Statistical Computing, Vienna, Austria, 2015). ANOVA was carried out using the “lm” function and Tukey’s test was carried out using the “HSD.test” function in the “agricolae” package. PCA was carried out after each experiment for various acrylamide concentrations considering bacteria and yeast separately; i.e., pH and temperatures. We considered those variables as scores and the numbers of microorganisms determined at a particular time of collecting samples as loadings. The PCA was carried out in “psych” package [32]. The Cortest-Bartlett test was carried out using the “cortest.bartlett” function in the “psych” package as well. The data demonstrated normal distribution so its transformation was not necessary. Strong correlations between loads and scores were considered when values obtained in correlation matrix exceeded 0.3 or −0.3.

Based on the basic statistics and results obtained in the PCA we selected optimum conditions for continuing experiments.

## 3. Results and Discussion

### 3.1. The Impact of Various Acrylamide Concentrations on Microbial Growth

In the current paper, we decided to verify whether yeast and/or bacteria, which naturally occur in food products or are used for food fermentation, could utilise or decompose acrylamide and therefore limits its negative impact on the human body. Therefore, we selected microorganisms which can naturally occur in fermented milk products (two strains of LAB and two strains of yeasts) and one bacterial strain that demonstrate probiotic properties (*L. acidophilus* LA-5).

The lactic acid bacteria *S. thermophilus* did not demonstrate growth in the MRD, the medium in which the carbon and nitrogen source are very limited (only 0.45% of bacteriological peptone), regardless of the absence or presence of acrylamide. In turn, *L. mesenteroides* DSM 20,343 showed weak growth in MRD, the number of cells decreased in all experimental variants (both with and without acrylamide) after 24 h and remained constant up to 48 h (Table 1). Although differences between the negative and positive control were not statistically significant, it can be seen that an additional 1 mL of MRD (in positive control) enhanced the *L. mesenteroides* growth, suggesting that bacteria were ready to quickly utilise any additional source of carbon and nitrogen. This also means that not only did MRD not provide enough nutrients for the basal metabolism of *L. mesenteroides*, but also that AA was not used as an additional carbon/nitrogen source by these bacteria. Contrary to *L. mesenteroides*, the number of *L. acidophilus* LA-5 cells was increasing with the concentration of acrylamide, reaching the maximum at 7.5 µg of AA/mL and it was significantly higher than all other tested variants, including positive control (Table 1). Therefore, *L. acidophilus* LA-5 has been chosen for further experiments.

When *S. cerevisiae* DSM 70,478 was considered, it was shown that after 24 h, the yeast number was equal between controls and experimental variants. This suggests that the presence of AA did not affect *S. cerevisiae* growth and indicates that the tested yeast is not able to utilise acrylamide. Moreover, the number of yeast cells decreased drastically after 48 h in all tested variants (to values below 3 × 10^4^ cfu/mL), indicating that the majority of nutrients present in MRD have already been exhausted (Table 1) and cells started to die. This would also mean that the MRD was the only source of available nutrients required for the tested strain. On the contrary, the growth of other tested yeast, *Kluyveromyces lactis* var. *lactis* DSM 70799, was enhanced when acrylamide concentration in culture equalled 10 µg/mL and reached the values similar to those of positive control. This suggests that acrylamide was utilised by *K. lactis* as an additional source of carbon and nitrogen allowing yeast to reproduce, so this strain was selected for further tests too.

**Figure 1:**
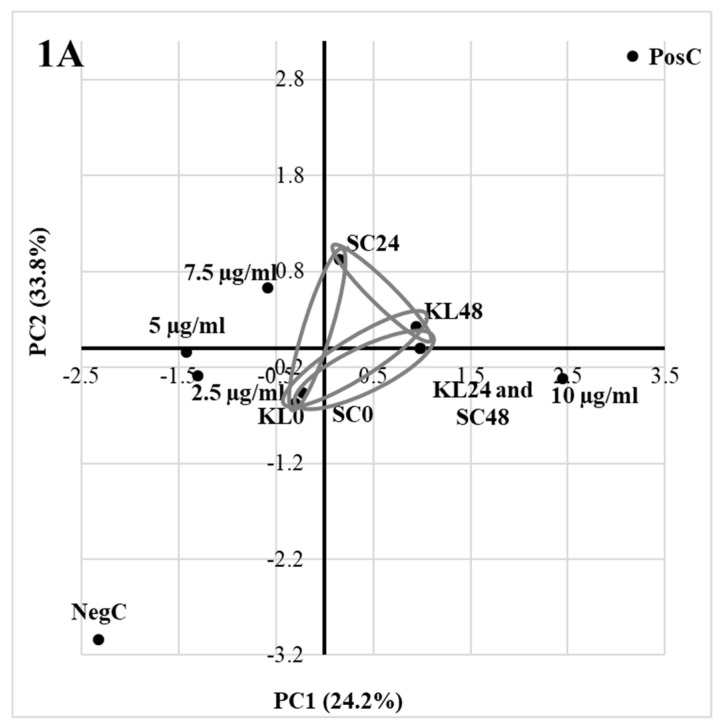

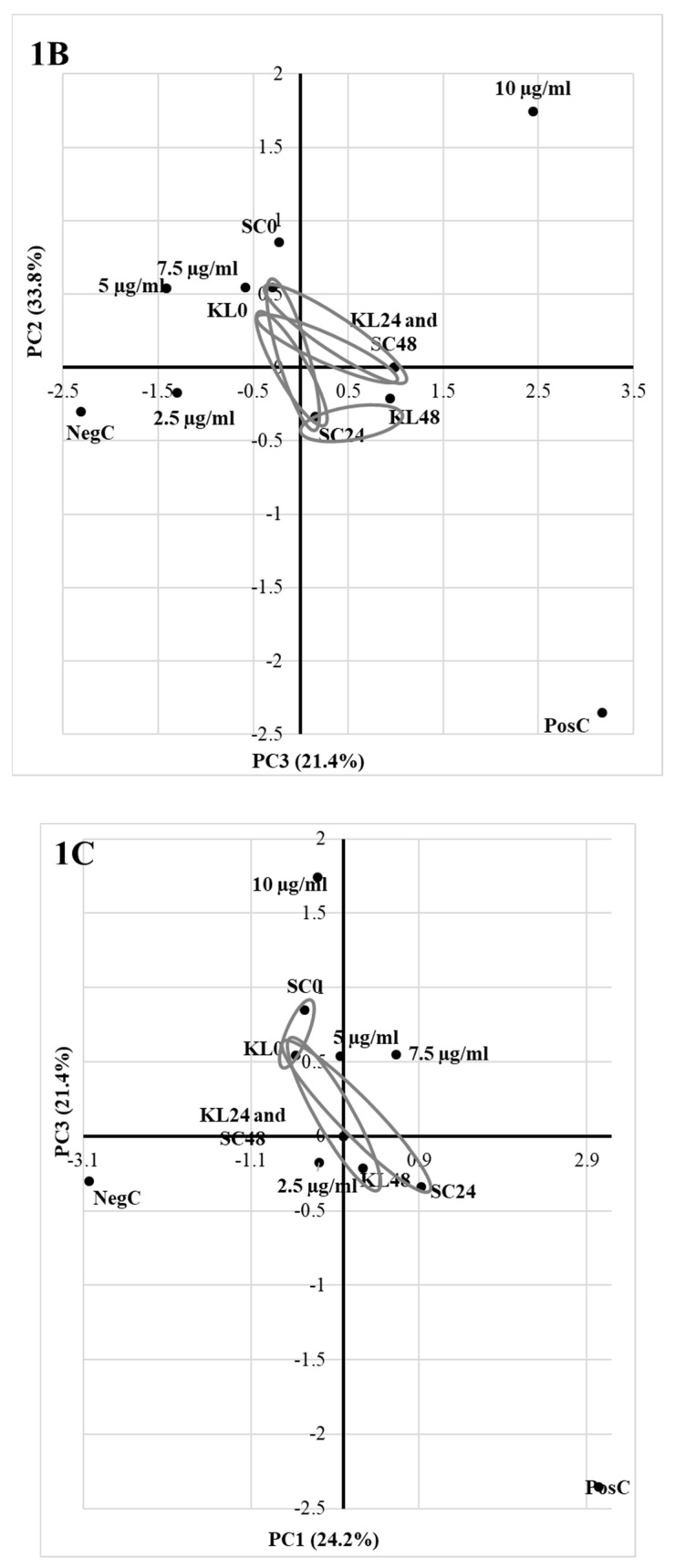
The PCA analysis of ammonia released at different times and initial acrylamide concentrations (scores) under various yeast strains–*Saccharomyces cerevisiae* DSM 70,478 and *Kluyveromyces lactis* var. *lactis* DSM 70,799 (loadings) for the first three components (**A**–**C**); circles indicate correlated loads and scores. Cneg, negative control; Cpos, positive control; KL0 and SC0, ammonia concentration at time 0; KL24 and SC24, ammonia concentration at time 24 h; KL48 and SC48, ammonia concentration at time 48 h.

It was only possible to carry out PCA for the results that were obtained in the experiment investigating the responses of both tested yeast strains to various acrylamide concentrations. In all other tested cases, the results of the Cortest-Bartlett test did not allow continuation of PCA. Based on PCA, in the case of *S. cerevisiae* and *K. lactis* we observed negative correlation between the number of cells in those strains determined at time “0”and after 24 h, respectively (Figure 1A–C).

It took place since the number of cells remained constant within 24 h for *S. cerevisiae*, while it increased for the latter yeast. Another negative correlation occurred for *K. lactis* at time “0”and 48 h. It was initially that the number of fungi was the same in all variants, while it varied after 48 h depending on the AA concentration. It was the greatest in positive control (C_pos_) and when the acrylamide concentration was 10 µg of AA/mL (Table 1). Another negative correlation (Figure 1C) indicated that an acrylamide concentration of 5 µg/mL was not sufficient to sustain *K. lactis* growth after 48 h.

### 3.2. The Impact of pH and Temperature on Microbial Growth in the Presence of Acrylamide

Based on the results from first experiment, for further analysis describing the impact of pH and temperature on the microorganisms’ growth in the presence of AA only *K. lactis* var. *lactis* and *L. acidophilus* LA-5 were selected.

Firstly, we chose a pH range that occurs within the human digestive tract (pH 2–9) and verified how it affects microbial growth without the addition of acrylamide. We noted that the selected bacterium strain was not affected at pH from 5 to 8, while the growth of fungal strain was unaffected from pH 4 to 9 (data not shown). Due to those findings, we continued experiments by adding acrylamide to the MRD adjusted to particular pH values. The acrylamide concentration that was used for this experiment was selected based on the results presented above (Table 1) as the acrylamide concentration that most strongly stimulated the growth of the test microorganisms (i.e., 7.5 µg AA/mL for *L. acidophilus* and 10 µg AA/mL for *K. lactis*).

We demonstrated that after 24 h at a pH of 4, 6, and 7, the presence of acrylamide in medium did not affect yeast number. However, at pH 7 it was slightly higher (lack of statistical significance) in the variant that included acrylamide than in control (Table 2). The growth of yeast at pH 8 and 9 was significantly reduced when compared to conditions closer to optimal. In turn, at pH 4 the presence of acrylamide allowed for maintaining similar number of cells after 24 and 48 h, while in the control sample the yeast concentration decreased significantly after 48 h. At pH 5, the addition of acrylamide caused slower fungal growth after 24 h, but after 48 h there were no statistically significant differences between control and MRD with AA (Table 2).

When the growth of probiotic *L. acidophilus* was analysed, it could be observed that even in poor medium (MRD containing only 0.45% peptone) the bacteria multiplied and their number increased in control samples at pH 5, 6, and 7 after 24 h of culture. However, after further 24 h it decreased, as all available nutrients were run out and bacteria start to die. Both after 24 h and 48 h of incubation, at all tested pH, the addition of acrylamide resulted in the increased number of bacterial cells in comparison to controls without acrylamide. The greatest bacteria count was demonstrated at pH 6, especially after 48 h (Table 2). In these samples acrylamide was an additional source of carbon and nitrogen, so when peptone components had finished bacteria could utilise acrylamide(after switching their metabolism) and still proliferate reaching the maximum count after 48 h. Taking into account that changing the metabolism pathway as well as utilising the acrylamide take time, the time shift of the peak of *L. acidophilus* LA-5 count is visible.

For determining the impact of temperature on the growth of tested microorganisms we chose pH 7 for *K. lactis* var. *lactis* and pH 6 for probiotic bacteria *L. acidophilus* LA-5. Three different temperatures were analysed: 4 °C (representing the temperature of the storage of fermented milk beverages), 20 °C (the mean room temperature), and 37 °C (the temperature of human body). We demonstrated that the most optimal temperature for microbial growth was 20 °C for yeast and 37 °C for bacterium (Table 3). However the differences between culture with and without acrylamide were not statistically significant, except for tests carried out at 37 °C after 48 h (Table 3). Both in the case of bacteria and yeast, at 37 °C the growth was better in the AA presence and the number of microorganisms was significantly higher than in control.

### 3.3. Utilisation of Acrylamide by Microorganisms

**Figure 2:**
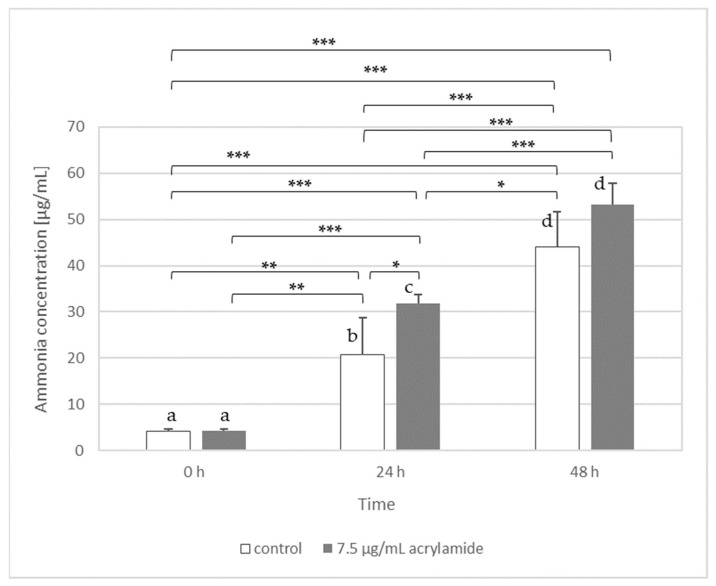
The production of ammonia by *L. acidophilus* LA-5 assessed in MRD medium without (control) or with 7.5 µg/mL of acrylamide after 24 h and 48 h of incubation with bacteria at 37 °C and pH 6. The same letters above bars indicate the lack of statistical differences. Significance level: * *p* < 0.05, ** *p* < 0.01, and *** *p* < 0.001.

For this part of experiment, we chose a probiotic strain *L. acidophilus* LA-5 and yeast *K. lactis* var. *lactis*. Our results have shown that only *L. acidophilus* LA-5 was able to release ammonia from acrylamide (Figure 2) and then use it as a source of nitrogen, while the rest of the molecule was probably introduced into the biochemical transformation of carbon-compounds. In the case of *K. lactis* var. *lactis* the ammonia concentration in samples with the addition of 10 µg AA/mL did not differ significantly from the control samples (data not shown).

Based on these results, we suggest that *L. acidophilus* LA-5 has the ability to amidase synthesis, which could be confirmed by showing that the content of ammonia (the most probably released from acrylamide by amidase) was statistically significantly higher in AA-containing medium inoculated with bacteria (after 24 h) than in control medium without AA. The ammonia can be also produced by bacteria from medium components or from cell compounds released to medium from bacteria after cell lysis (e.g., from amino acids) [33]. This process can explain why after 48 h the level of ammonia still increased in all samples (both with and without acrylamide). However, the level of detected ammonia was higher in samples with acrylamide, suggesting that amidases were also involved in this process.

The results presented in Figure 2 suggest that there was a sufficient ammonia concentration as a nitrogen source in both control medium and the AA-containing medium at 24 h. The factor that limited the *L. acidophilus* LA-5 growth within the next 24 h was probably the availability of a carbon source. We suppose that bacteria firstly (within 24 h) utilised the easily available carbon/nitrogen sources (i.e., peptone), both in samples with and without acrylamide. Then, in the control medium, due to the depletion of a carbon source, the number of *L. acidophilus* LA-5 could not increase from 24 h to 48 h (Table 3). In medium with acrylamide, the bacteria had to switch their metabolism. Thanks to that, *L. acidophilus* LA-5 could utilise acrylamide as a carbon source and the bacteria number could further increase from 24 h to 48 h (Table 3). The greater number of bacterial cells (in medium with AA) implicates that-even if the percentage of dying cells remains unchanged-the absolute number of the dead cells is higher. As a result, more components of lysed cells are released into the medium, from which ammonia could be produced (the higher ammonia concentration in medium both with and without AA after 48 h versus 24 h in Figure 2).

It should be highlighted that the number of bacteria (expressed as cfu/mL) cannot be directly translated into the amount of ammonium released. The “cfu” reflects the number of bacteria that can multiply and form colonies. Moreover, not all cells had been able to switch their metabolism and utilise acrylamide. As can be seen in Table 3., the number of *L. acidophilus* LA-5 in the medium with AA after 48 h of incubation at 37 °C was three-fold higher than in the control. On the other side, the amount of ammonia released by *L. acidophilus* LA-5 in the experiment conducted under the same conditions (Figure 2) increased by about 1.2 times after 24 h, while after 48 h the difference was no longer statistically significant. In our opinion, it is due to the fact that the concentration of AA used was low (7.5 µg/mL) and all AA was quickly used up by growing bacteria as available nutrient. Hence, after 48 h no further ammonia was released from acrylamide. After the acrylamide resource had depleted, the growth limiting factor seems to be the lack of an available carbon source, and therefore the number of cells did not increase proportionally. It should also be recalled that results presented in Table 2 and Table 3 as well as Figure 2 come from independent experiments. Each of them was performed five replicates, and the results are expressed as arithmetic mean, but the analyses (cell number and ammonia concentration) were not conducted in the same sample of medium.

It is also interesting that, taking into account the reaction stoichiometry, one mole of acrylamide amidase can release one mole of ammonia. It means that addition of 7.5 µg of acrylamide could theoretically generate about 1.8 µg of ammonia if the whole acrylamide was used in the reaction. As we can see in Figure 2, the amount of ammonia determined in the experiment, and actually the difference between the control sample and medium with acrylamide, is higher than the value resulting solely from the presence and decomposition of acrylamide. In cell free samples, both with and without AA, the ammonia concentration was below the detection level of the assay (therefore appropriate bars were not shown in the Figure 2), suggesting that ammonia in the medium could not be produced during autoclave sterilization. The results proved that ammonia in samples was produced only in the presence of microorganism; it could not be generated spontaneously from the MRD components (amino acids of peptone) regardless of the pH and temperature used. This, however, does not exclude that ammonia could be also produced by bacteria enzymes from cell compounds released to medium after cell lysis (e.g., from amino acids).

There are several ways to reduce the acrylamide content in food products. For example, the formation of acrylamide can be reduced by selecting appropriate vegetable varieties (poor in acrylamide precursors such as asparagine and reducing sugars), by limiting the time and temperature during thermal processing, as well as by using an appropriate frying media or antioxidants [8,34]. It is also possible to use the lactic acid fermentation before frying, e.g., *Lactobacillus plantarum* fermentation reduced the simple sugars content in potato rods, which allows to decrease the acrylamide formation in French fries [35]. It was also reported that the application of selected strains of LAB or the usage of enzymes that catalyse deamination of asparagine and glutamine (L-asparaginase and L-glutaminase, respectively) allowed for a significant decrease of acrylamide formation [36,37,38]. Esfahani et al. [39] proved that the reduction of acrylamide content of whole-wheat breads can be obtained by combining lactobacilli and yeast in sourdough fermentation, since those microorganisms use precursors of acrylamide as a source of carbon and nitrogen. It has also been reported that acrylamide can be decomposed under the influence of microbial amidases resulting in the release of ammonia and acrylic acid, which might be then transformed in various pathways to β-hydroxypropionate, propionate, lactate or CO_2_. However, the studies were focused on environmental bacteria species and not lactic acid bacteria [40,41,42].

The ability to amidases synthesis has been already demonstrated in various bacteria, including LAB. For example, the synthesis of N-acetylmuramoyl-L-alanine amidase, involved in the degradation of peptidoglycan in the bacterial cell wall [43], was reported in *Lactobacillus sakei*, as well as the synthesis of N-acetylmuramidase with a key role in autolysis was demonstrated for *Lactobacillus bulgaricus* [44]. Amidases with activity of murein hydrolase are produced by staphylococci and are necessary for the generation of the equatorial ring on the cell surface and complete cell division and separation [45]. Similarly, the division of *Escherichia coli* depends on the activity of amidases AmiA, AmiB, and AmiC [27]. However, there are no published papers demonstrating that amidases produced by lactic acid bacteria can utilise acrylamide as a substrate for deamination. Our results, for the first time, report that the probiotic strain *Lactobacillus acidophilus* LA-5 has the ability to decompose acrylamide and to release ammonia, which can prove that acrylamide might be another substrate for amidases. Taking into account the results of experiments, especially that the bacteria count after 48 h in samples with acrylamide was higher than in those without acrylamide (Table 2 and Table 3), as well as fact that the excess of ammonia (difference in ammonia concentration between samples with and without acrylamide reported after 24 h–Figure 2) had “disappeared” in 48 h, we conclude that the released ammonia had been utilised by bacteria as a source of nitrogen, while the rest of the molecule (acrylic acid or its metabolites) as a carbon source. Of course, our experiments were conducted in a medium with very limited carbon and nitrogen sources, so further experiments are planned to confirm whether *L. acidophilus* will retain the ability to acrylamide utilisation in the environment where various nutritional compounds and substrates are easily available.

Nevertheless, our studies have shown the possibility that some LAB strains could exert beneficial impact on human organisms after intestine colonisation, not only by their well-known impact on digestion and maintenance of proper gastrointestinal balance, but also by acrylamide utilisation and preventing in such way against its harmful impact on the human body.

### 3.4. Response Surface Methodology

Since we demonstrated that only *L. acidophilus* LA-5 was a putative amidase producer, that strain was selected for the experiments involving RSM. We considered variables that significantly influenced the growth of mentioned bacterium in the presence of acrylamide: pH (X_1_), temperature (X_2_) and time (X_3_). In our study, RSM was performed to avoid carrying out dozens of experiments in a very broad range of tested variables: temperature 4–37 °C, pH 5–7, and time 0–48 h. The response in the model was ammonia concentration (µg/mL) measured at particular experimental time. For the first run of experiments for central point we decided to apply the following values: pH 6.0, temperature 20 °C, and time 24 h (Table 4). As increments we selected the following values: 1 for pH, 17 for temperature and 24 for time. We demonstrated that only first order model explains investigated phenomenon with satisfactory reliability (R^2^ = 0.9807 and adjusted R^2^ = 0.9663). After the first and second run of experiments we have moved centre points to: pH 6.78, temperature 33 °C, and time 48 h. This operation caused significant decrease in R^2^ and adjusted R^2^ (0.02244 and −0.7107, respectively, for experimental run 3; 0.02441 and –0.3303, respectively for experimental run 4, Table 4), and therefore we concluded that first order model could be reliable only in the range of pH 6 to 7, temperature 20 to 37 °C and time 24 to 48 h. When we tested if the data could fit to the second order model it was proved to fail, since R^2^ and adjusted R^2^ were too low (0.6914 and 0.4084, respectively, for experimental run 5, Table 4). Those figures did not improve when we changed central points to: pH 6.67, temperature 34.7 °C, and time 75 h.

When we tested cube blocks of the model, we obtained R^2^ = 0.8224 and adjusted R^2^ = 0.4672. That improvement was not sufficient to provide reliable results. Moreover, based on the analysis of that model, centre points were: pH = 3.25, temperature = 52.98 °C, and time = 107.2 h. Those figures could lead to false conclusions since at those conditions, bacterial cells could be disintegrated which could lead to the release of a significant ammonia amount. Due to this fact, we did not present contour plots (response surface plots) in the current paper.

Therefore, the final version of the model includes only first order and is:

(2) $$Y=9.55+1.204X_{1}+0.254X_{2}+0.227X_{3}$$

**Figure 3:**
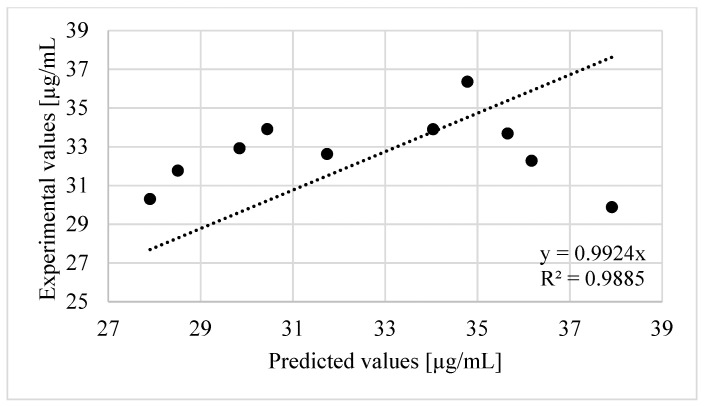
Validation of the model describing ammonia release built with RSM–regression analysis of experimental and predicted values.

At the end, the validation of the model built with RSM was carried out within pH 6 to 7, temperature 20 to 37 °C and time 24 to 48 h (Table 5). The obtained value of regression coefficient (R^2^ = 0.9885) between predicted values and experimental values of ammonia concentration (Figure 3) had confirmed that Equation (2) is well suited.

Our results could be important not only in food production. Bacteria after colonization of human gut could use up acrylamide and therefore detoxify it just directly in the human body. Especially since it turned out that the most favourable temperature is 37 °C, so the same as in the intestine. In conclusion, thanks to using RSM, we could obtain a mathematical equation which enables a prediction of released ammonia by *L. acidophilus* LA-5. It also suggests more narrow ranges of variables that should receive special attention in further studies. In overall, the results of present research justify further studies, especially in the food matrix.

## 4. Conclusions

Acrylamide present in food product can exert both a positive and negative impact on intestinal microbiota. Fortunately, some bacteria strains, including beneficial LAB, are resistant to the presence of this toxic substance, and what is more important is that they can use acrylamide as an additional source of carbon and nitrogen in a situation where the environment (medium) is lacking for them. To the best of our knowledge, we showed for the first time that the probiotic strain of bacteria *L. acidophilus* LA-5, often added to yogurts by food producers, has the ability to degrade acrylamide and used the ammonia released by amidase as a source of nitrogen and the rest of molecule (acrylic acid or its metabolites) as a carbon source. The analysis of the variance and RSM indicated that pH as well as incubation time and temperature significantly influenced the amount of ammonia released from acrylamide by bacteria.

Our studies suggest that some strains of bacteria present in milk fermented products, especially yoghurts, can exert a beneficial impact on human organisms, not only by their well-known impact on digestion and maintenance of proper gastrointestinal balance, but also by utilisation of acrylamide and helping prevent against its harmful impact on the human body. These LAB abilities may become of particular importance when acrylamide is added to non-heat treated food with additives such as muesli, roasted nuts, high-temperature dried fruit, seeds, coffee, etc., often after the fermentation process. Bacteria are still alive and need nutrients and it is possible that acrylamide can be used up by them while still in yoghurt. Additionally, demonstrating that lactic acid bacteria, when consumed with fermented foods, have the ability to reduce the concentration of acrylamide in the human gastrointestinal tract is extremely important. Therefore, further studies are required to show whether the ability of acrylamide utilisation is carried out by intestinal bacteria in the colon milieu, where the concentration of nutrients is high. Moreover, the impact of acrylamide on the metabolism pathways of lactic acid bacteria should be also checked.

## Figures and Tables

**Table 1:** The impact of various acrylamide (AA) concentrations on the number of selected bacteria and yeast in maximum recovery diluent (MRD) medium after incubation (n = 5).

Incubation Time	C_neg_	AA 2.5 µg/mL	AA 5.0 µg/mL	AA 7.5 µg/mL	AA 10 µg/mL	C_pos_
*Leuconostoc mesenteroides* subsp. *cremoris* DSM 20346 [×10^6^ cfu/mL]						
0 h	10.4 ± 11.4 abc	18.0 ± 7.8 ab	18.3 ± 8.8 ab	8.8 ± 9.9 abc	23.1 ± 11.0 a	19.7 ± 1.7 ab
24 h	1.2 ± 0.5 c	1.2 ± 0.3 c	1.5 ± 0.4 c	1.3 ± 0.6 c	2.1 ± 0.5 c	2.54 ± 0.8 c
48 h	1.4 ± 0.2 c	2.3 ± 0.7 c	1.9 ± 0.3 c	1.5 ± 0.5 c	2.3 ± 0.2 c	1.16 ± 0.6 c
*Lactobacillus acidophilus* LA-5 [×10^6^ cfu/mL]						
0 h	2.6 ± 1.0 cd	2.4 ± 0.5 cd	2.9 ± 0.2 cd	1.62 ± 0.25 d	1.8 ± 0.7 d	2.0 ± 0.8 d
24 h	2.62 ± 1.3 cd	11.8 ± 3.2 bc	51.8 ± 5.4 ab	65.4 ± 47.0 a	7.2 ± 2.2 cd	32.6 ± 0.9 abc
48 h	6.0 ± 7.6 cd	1.0 ± 0.0 d	2.0 ± 0.7 d	46.3 ± 17.6 ab	19.6 ± 11.1 bc	2.6 ± 0.9 cd
*Saccharomyces cerevisiae* DSM 70478 [×10^4^ cfu/mL]						
0 h	7.6 ± 1.3 ab	5.8 ± 1.0 ab	6.7 ± 1.2 ab	6.0 ± 1.3 ab	6.4 ± 1.3 ab	5.5 ± 1.3 ab
24 h	4.8 ± 4.9 ab	7.2 ± 2.4 ab	9.0 ± 2.6 a	8.4 ± 2.0 ab	7.2 ± 1.1 ab	9.8 ± 1.1 a
48 h	<3.0 ± 0.0 c	<3.0 ± 0.0 c	<3.0 ± 0.0 c	<3.0 ± 0.0 c	<3.0 ± 0.0 c	<3.0 ± 0.0 c
*Kluyveromyces lactis* var. *lactis* DSM 70799 [×10^5^ cfu/mL]						
0 h	4.7 ± 1.4 b	3.7 ± 0.5 b	4.2 ± 0.7 b	2.8 ± 0.6 b	4.4 ± 1.3 b	4.6 ± 0.8 b
24 h	4.8 ± 0.8 b	5.0 ± 0.8 b	5.1 ± 1.4 b	4.64 ± 1.1 b	17.2 ± 8.2 a	14.2 ± 2.3 a
48 h	3.8 ± 1.3 b	6.4 ± 3.8 ab	3.8 ± 2.6 b	5.2 ± 4.2 b	9.0 ± 5.3 ab	9.6 ± 4.0 ab

The same letters below the values within the results for particular microorganism indicate the lack of statistical differences at *p* < 0.05. C_pos_–positive control (9 mL of MRD inoculated with 1 mL microbial suspension), C_neg_–negative control (8 mL of MRD inoculated with 1 mL of microbial suspension and 1 mL of sterile water). For DSM 70,478 the number of yeast colonies after 48 h was <30 on the plate or they were not detected at all. Thus, the results are presented as <3.0 ± 0.0 (the lowest sample dilution spread on the plate with YM medium was 10^−3^).

**Table 2:** The impact of pH on the growth of *Kluyveromyces lactis* var. *lactis* and *Lactobacillus acidophilus* LA-5 in the presence of acrylamide (AA) (n = 5).

***Lactobacillus acidophilus* LA-5 [×10^5^ cfu/mL]** **(Incubation at 37 °C, 7.5 µg AA/mL)**							
Time	Sample	pH 4	pH 5	pH 6	pH 7	pH 8	pH 9
0 h	Control	NA	2.3 ± 0.5 e	2.5 ± 0.7 f	4.3 ± 0.9 e	3.7 ± 0.7 e	NA
	AA	NA	6.6 ± 1.1 d	8.0 ± 1.4 d	2.6 ± 0.2 f	3.7 ± 1.0 e	NA
24 h	Control	NA	9.1 ± 1.40 bc	6.3 ± 1.9 d	5.0 ± 0.5 e	<0.01 i	NA
	AA	NA	9.2 ± 2.8 bc	12.1 ± 1.9 b	7.1 ± 0.9 d	1.3 ± 0.2 b	NA
48 h	Control	NA	0.6 ± 0.2 h	2.3 ± 0.4 f	1.0 ± 0.5 g	<0.01 i	NA
	AA	NA	1.3 ± 0.2 g	>30 a	5.3 ± 1.3 d	9.5 ± 1.7 bc	NA
***Kluyveromyces lactis* var. *lactis* [×10^5^ cfu/mL]** **(incubation at 28 °C, 10 µg AA/mL)**							
Time	Sample	pH 4	pH 5	pH 6	pH 7	pH 8	pH 9
0 h	Control	6.6 ± 1.0 d	5.2 ± 0.6 d	6.4 ± 1.1 d	6.2 ± 1.1 d	5.3 ± 0.4 d	5.6 ± 1.0 d
	AA	7.3 ± 0.7 d	6.5 ± 1.0 d	8.3 ± 1.3 d	7.8 ± 1.4 d	6.5 ± 1.0 d	6.9 ± 0.7 d
24 h	Control	14.8 ± 4.6 a	12.8 ± 3.6 a	13.2 ± 2.7 a	13.6 ± 1.3 a	3.2 ± 0.9 e	0.7 ± 0.2 f
	AA	12.4 ± 2.3 a	9.4 ± 0.3 c	13.8 ± 1.9 a	18.0 ± 5.6 a	0.4 ± 0.2 f	2.9 ± 0.6 e
48 h	Control	7.5 ± 1.4 d	13.9 ± 0.3 a	11.2 ± 1.1 ab	12.1 ± 1.7 ab	5.4 ± 2.3 de	0.9 ± 0.2 f
	AA	16.3 ± 2.1 a	13.2 ± 1.8 a	12.7 ± 1.5 ab	10.9 ± 1.2 b	5.2 ± 0.7 de	0.7 ± 0.1 f

The same letters below the arithmetic means for each microorganism indicate the lack of statistical differences at *p* < 0.05. Abbreviations: AA, acrylamide present in MRD medium at concentration of 10 µg/mL (yeast) or 7.5 µg/mL (bacteria); Control, control sample prepared by adding 1 mL of microbial suspension to 9 mL of AA-free MRD adjusted to adequate pH; NA, not analysed.

**Table 3:** The impact of temperature on the growth of *Kluyveromyces lactis* var. *lactis*, and *Lactobacillus acidophilus* LA-5 in the presence of acrylamide (AA) (n = 5).

***Lactobacillus acidophilus* LA-5 [×10^5^ cfu/mL]** **(pH 6, 7.5 µg AA/mL)**				
Time	Sample	4 °C	20 °C	37 °C
0 h	Control	4.0 ± 0.7 bc	4.8 ± 1.0 bc	3.7 ± 0.7 bc
	AA	3.9 ± 0.6 bc	4.8 ± 1.3 bc	4.5 ± 1.1 bc
24 h	Control	3.7 ± 0.6 bc	5.3 ± 0.8 b	4.8 ± 1.6 bc
	AA	3.5 ± 0.7 bc	4.9 ± 1.6 bc	6.3 ± 1.5 b
48 h	Control	3.3 ± 0.8 bc	1.9 ± 0.68 c	5.7 ± 0.6 b
	AA	2.8 ± 0.4 bc	2.6 ± 0.6 bc	17.0 ± 7.1 a
***Kluyveromyces lactis* var. *lactis* [×10^5^ cfu/mL]** **(pH 7, 10 µg AA/mL)**				
Time	Sample	4 °C	20 °C	37 °C
0 h	Control	1.4 ± 0.1 c	1.6 ± 0.1 c	1.2 ± 0.2 c
	AA	1.5 ± 0.4 c	1.4 ± 0.3 c	1.4 ± 0.3 c
24 h	Control	1.8 ± 0.6 c	6.8 ± 1.4 b	0.8 ± 0.1 c
	AA	2.2 ± 0.3 c	7.1 ± 1.6 b	1.0 ± 0.1 c
48 h	Control	1.6 ± 0.3 c	21.7 ± 3.3 a	1.4 ± 0.4 c
	AA	1.9 ± 0.3 c	19.7 ± 0.9 a	8.2 ± 2.3 b

The same letters below the arithmetic means for each microorganism indicate the lack of statistical differences at *p* < 0.05. Abbreviations: AA, acrylamide present in MRD medium at concentration of 10 µg/mL (yeast) or 7.5 µg/mL (bacteria); Control, control sample prepared by adding 1 mL of microbial suspension to 9 mL of AA-free MRD adjusted to adequate pH.

**Table 4:** The summary of response surface methodology carried out against *Lactobacillus acidophilus* LA-5 to examine its ability to produce ammonia in the presence of acrylamide. The table present Box-Behnken design matrix and response values.

Experimental Run	Coded Variables			Actual Variables			Ammonia Conc. [µg/mL]
	X_1_	X_2_	X_3_	pH	Temperature [°C]	Time [h]	
1	−1	1	−1	5	37	0	0
	1	−1	−1	7	3	0	0
	0.0	0	0	6	20	24	10.20
	−1	−1	1	5	3	48	4.90
	0	0	0	6	20	24	7.80
	1	1	1	7	37	48	38.60
	0	0	0	6	20	24	6.70
	0	0	0	6	20	24	8.20
2	1.50	1.57	2.02	7.57	46.64	72.53	1.00
	1.05	1.05	1.35	7.05	37.77	56.35	16.25
	0.78	0.78	1.01	6.78	33.31	48.26	11.48
	0.52	0.52	0.67	6.52	28.87	40.18	3.66
	1.30	1.31	1.69	7.31	42.20	64.44	2.16
	0.26	0.26	0.34	6.26	24.44	32.09	2.45
3	1.61	1.68	2.01	7.78	50	72	4.05
	1.40	1.11	1.34	6.78	33	48	13.36
	1.61	0.54	0.67	7.78	16	24	4.24
	1.40	1.11	1.34	6.78	33	48	13.18
	1.20	0.54	2.01	5.78	16	72	5.98
	1.20	1.68	0.67	5.78	50	24	5.79
	1.40	1.11	1.34	6.78	33	48	13.18
	1.40	1.11	1.34	6.78	33	48	13.01
4	1.20	0.54	0.67	5.78	16	24	4.33
	1.61	1.68	0.67	7.78	50	24	3.88
	1.40	1.11	1.34	6.78	33	48	15.05
	1.61	0.54	2.01	7.78	16	72	4.65
	1.20	1.68	2.01	5.78	50	72	6.26
	1.40	1.11	1.34	6.78	33	48	15.49
	1.40	1.11	1.34	6.78	33	48	15.49
	1.40	1.11	1.34	6.78	33	48	15.94
5	1.40	1.11	2.28	6.78	33.00	81.94	29.17
	1.11	1.11	1.34	5.37	33.00	48.00	10.50
	1.70	1.11	1.34	8.19	33.00	48.00	6.82
	1.40	1.11	1.34	6.78	33.00	48.00	10.43
	1.40	1.92	1.34	6.78	57.04	48.00	9.78
	1.40	1.11	0.39	6.78	33.00	14.06	2.14
	1.40	1.11	1.34	6.78	33.00	48.00	19.61
	1.40	0.30	1.34	6.78	8.96	48.00	4.63
6	−0.137	0.04	1.708	6.643	33.68	88.992	27.1
	−0.078	0.041	0.565	6.702	33.697	61.56	26.67
	−0.126	0.043	1.422	6.654	33.731	82.128	27.65
	−0.158	0.032	2.28	6.622	33.544	102.72	26.75
	−0.148	0.036	1.994	6.632	33.612	95.856	25.11
	−0.113	0.044	1.136	6.667	33.748	75.264	29.05
	−0.097	0.044	0.85	6.683	33.748	68.4	27.5
7	1.17	0.59	1.42	5.67	17.7	51	8.56
	1.59	1.74	2.76	7.67	51.7	99	9.65
	1.17	1.74	2.76	5.67	51.7	99	7.70
	1.59	0.59	2.76	7.67	17.7	99	18.23
	1.17	0.59	2.76	5.67	17.7	99	8.51
	1.17	1.74	1.42	5.67	51.7	51	10.06
	1.59	0.59	1.42	7.67	17.7	51	14.11
	1.59	1.74	1.42	7.67	51.7	51	5.47
	1.38	1.17	2.09	6.67	34.7	75	24.56
	1.38	1.17	0.75	6.67	34.7	27	19.62
	1.38	1.17	3.43	6.67	34.7	123	27.51
	1.38	1.17	2.09	6.67	34.7	75	12.08
	1.38	0.02	2.09	6.67	0.7	75	1.95
	1.80	1.17	2.09	8.67	34.7	75	27.55
	0.97	1.17	2.09	4.67	34.7	75	11.63
	1.38	2.31	2.09	6.67	68.7	75	8.47

**Table 5:** Experimental variants used for the validation of the model.

pH	Temperature [°C]	Time [h]	Predicted Values [µg/mL]	Experimental Values [µg/mL]
6.1	33	48	36.17	32.27
6.7	37	48	37.91	29.87
6.5	30	24	30.44	33.90
6.5	20	24	27.90	30.29
6	28	48	34.78	36.35
7	20	24	28.51	31.76
6	30	24	29.84	32.92
6.7	28.9	40	34.04	33.90
6.3	30	48	35.65	33.68
6.1	37	24	31.74	32.62

Predicted and experimental values refer to ammonia concentrations.

## Data Availability

The data presented in this study are available on request from the corresponding author.
